# Genetic Dissection of Epidermal Growth Factor Receptor Signaling during Luteinizing Hormone-Induced Oocyte Maturation

**DOI:** 10.1371/journal.pone.0021574

**Published:** 2011-06-30

**Authors:** Minnie Hsieh, Kao Thao, Marco Conti

**Affiliations:** Department of Obstetrics, Gynecology & Reproductive Sciences, University of California San Francisco, San Francisco, California, United States of America; Universitat de Barcelona, Spain

## Abstract

Recent evidence that luteinizing hormone (LH) stimulation of ovulatory follicles causes transactivation of the epidermal growth factor receptor (EGFR) has provided insights into the mechanisms of ovulation. However, the complete array of signals that promote oocyte reentry into the meiotic cell cycle in the follicle are still incompletely understood. To elucidate the signaling downstream of EGFR involved in oocyte maturation, we have investigated the LH responses in granulosa cells with targeted ablation of EGFR. Oocyte maturation and ovulation is disrupted when EGFR expression is progressively reduced. In granulosa cells from mice with either global or granulosa cell-specific disruption of EGFR signaling, LH-induced phosphorylation of MAPK3/1, p38MAPK, and connexin-43 is impaired. Although the LH-induced decrease in cGMP is EGFR-dependent in wild type follicles, LH still induces a decrease in cGMP in *Egfr^delta/f^ Cyp19-Cre* follicles. Thus compensatory mechanisms appear activated in the mutant. Spatial propagation of the LH signal in the follicle also is dependent on the EGF network, and likely is important for the control of signaling to the oocyte. Thus, multiple signals and redundant pathways contribute to regulating oocyte reentry into the cell cycle.

## Introduction

In the preovulatory follicle, oocytes are arrested in prophase I of meiosis until the luteinizing hormone (LH) surge triggers oocyte meiotic resumption [1], [2], [3]. LH activates its G-protein-coupled receptor on theca and granulosa cells, leading to elevation of intracellular cAMP levels and branching of signals to multiple downstream pathways. Because cumulus cells and oocytes are insensitive to direct LH stimulation due to lack of LH receptors [4], [5], the LH effects on the cumulus-oocyte complex (COC) are thought to be mediated by paracrine factors and/or transfer of signals through gap junctions between follicular cells. By these means, LH-induced signals propagate from the mural granulosa cells to the COC and resumption of oocyte meiosis is induced.

Epidermal growth factor (EGF)-like growth factors are likely mediators of LH action in the preovulatory follicle [6], [7], [8]. They are expressed as integral membrane proteins that are shed from the cell surface as mature soluble factors and bind to members of the EGF receptor (EGFR, also called ErbB1) family, leading to receptor dimerization and autophosphorylation, and activation of downstream signaling cascades including the MAPK3/1 and AKT pathways [9], [10]. The EGF-like growth factors amphiregulin (AREG), epiregulin (EREG) and betacellulin (BTC) are rapidly induced in granulosa cells by LH or its analog human chorionic gonadotropin (hCG), and promote many events induced by LH, including cumulus expansion and oocyte maturation [6], [7], [11]. EGFR activity is required, as both the LH and growth factor effects are blocked by EGFR tyrosine kinase inhibitors [6], [7]. Mutant mice with disruption of the EGF signaling network (*Areg^−/−^ Egfr^wa2/wa2^*) have defects in cumulus expansion, oocyte maturation and ovulation, underscoring an important role of the EGF network in these LH-regulated processes [8]. Because LH transactivation of the EGFR is rapid and precedes the onset of oocyte maturation, this may serve as an important signal for oocyte meiotic resumption [12].

The steps involved in the induction of oocyte maturation are not completely understood. It has been shown that high levels of intra-oocyte cAMP, produced by G_s_-coupled receptor GPR3 activation of adenylyl cyclase, are essential for maintaining the meiotic arrest [13], [14], [15], [16], [17], [18]. A decrease in cAMP due to phosphodiesterase type 3A (PDE3A) activity in the oocyte allows meiosis to resume [19], [20]. Recent studies suggest that cGMP cooperates with cAMP in maintaining meiotic arrest by inhibiting PDE3A [21], [22]. LH induces a marked decrease in cGMP, which involves activation of the EGF network and results in relief of PDE3A inhibition [21], [22]. Gap junctions are proposed to regulate the passage of the inhibitory cGMP signal from the somatic cells to the oocyte [21]; gap junction inhibitors or removal of the COC from the follicle results in oocyte meiotic resumption [23], [24], [25]. Recently, LH was shown to induce the closure of gap junctions specifically between the somatic cells of the follicle, prior to the onset of oocyte meiotic resumption, via MAPK3/1-dependent phosphorylation of gap junction alpha-1 protein (GJA1, also called and referred to as connexin 43 or Cx43 herein) on serines 255, 262 and 279/282 [26]. Taken together, the LH-induced decrease in cGMP and gap junction closure in the somatic compartment contribute to the activation of PDE3A in the oocyte, leading to decreased oocyte cAMP and stimulation of meiotic resumption [19], [20], [21], [22], [26].

It is likely that additional mechanisms are required for the control of oocyte meiotic resumption. Granulosa cell-specific disruption of *MAPK3/1* (ERK1/2) revealed it to be necessary for oocyte maturation [27]. However, reduced but measureable levels of phosphorylated MAPK3/1 were detected in *Areg ^−/−^ Egfr^wa2/wa2^* mouse follicles in which oocyte maturation is impaired [12]. Also, the MEK1 inhibitor U0126 (10 µM) prevented MAPK3/1 activation and LH-induced Cx43 gap junction closure, but caused little inhibition, if any, of LH-induced oocyte maturation [26]. Although the *Areg^−/−^ Egfr^wa2/wa2 wa2^* mice that we previously generated have been important in demonstrating a role for the EGF network in oocyte maturation and ovulation, EGFR signaling is not completely ablated and extragonadal effects may contribute to impaired fertility in adult females [8], [28]. To circumvent this issue, we generated mice with granulosa cell-specific disruption of the *Egfr* (*Egfr^delta/f^ Cyp19-Cre*). To gain insight into how the EGF network mediates LH-induced oocyte maturation, we used both the *Areg^−/−^ Egfr^wa2/wa2 wa2^* and *Egfr^delta/f^ Cyp19-Cre* genetic mouse models and the preovulatory follicle culture model to examine the regulation of known targets of LH and EGFR signaling. Our findings demonstrate that EGFR is essential for efficient LH activation of oocyte maturation and ovulation and indicate that complex mechanisms regulated by both LH and EGFR act together or in parallel to promote the development of a mature, fertilizable egg.

## Materials and Methods

### Ethics Statement

All animal procedures were approved and followed the guidelines of the Institutional Animal Care and Use Committee at the University of California San Francisco.

### Materials

Recombinant LH (rLH, Luveris) was purchased from Serono International (Rockland, MA). Recombinant AREG was from R&D Systems, Inc. (Minneapolis, MN). Pregnant Mare Serum Gonadotropin (PMSG), AG1478 and SB202190 were purchased from Calbiochem (San Diego, CA). Penicillin-streptomycin solution and the inhibitor U0126 were from Sigma-Aldrich (St. Louis, MO). Complete, mini EDTA-free protease inhibitor cocktail tablets were purchased from Roche Applied Science (Indianapolis, IN). ECL and ECL Plus Western Blotting detection reagents were from Amersham Biosciences (Piscataway, NJ). Anti-ERK1 and anti-connexin 43 were from BD Biosciences (San Jose, CA), anti-phospho-MAPK (p44/42), anti-phospho-AKT, anti-AKT, anti-phospho-p38MAPK and anti-p38MAPK antibodies from Cell Signaling Technology (Danvers, MA), and anti-phospho-connexin 43 (serine 262), anti-phospho-connexin 43 (serine 255) and anti-EGFR antibodies from Santa Cruz Biotechnology (Santa Cruz, CA). Leibowitz's L15 medium, Minimum Essential Medium (MEM), Dulbecco's phosphate buffered saline (DPBS) without Ca^2+^ and Mg^2+^, and fetal bovine serum (FBS), were obtained from Gibco (Carlsbad, CA). UltraPure water and cyclic GMP EIA kits were purchased from Cayman Chemical Company (Ann Arbor, MI).

### Animals and hormone treatments

Immature (22–23 days old) female C57BL/6 mice were purchased from Charles River Laboratories (Wilmington, MA). *Areg^+/+^ Egfr^+/+^, Areg^+/+^ Egfr^wa2/+^* and *Areg^−/−^ Egfr^wa2/^* ^wa2^ mice were generated and genotyped as previously described [8]. Mice carrying the *Egfr^f^* allele [29] and the *Egfr^delta^* null allele were generously provided by David W. Threadgill. The *Egfr^f^* allele was detected by PCR using the lox3s and lox3as primers as described in Lee and Threadgill [29]. The Delta-3 and Delta-4 primers described in Lee and Threadgill [29] were used to detect the Cre-reduced *Egfr* allele in that study, but also are used to detect the *Egfr^delta^* null allele. The *Cyp19-Cre* mice were from JoAnne S. Richards [30] and were genotyped by PCR using the following primers: Cyp19Cre-F (5′-ACTTGGTCAAAGTCAGTGCG-3′) and Cyp19Cre-R (5′-CCTGGTGCAAGCTGAACAAC-3′). Mouse crosses were performed to generate *Egfr^f/f^ Cyp19-Cre*, *Egfr^delta/f^ Cyp19-Cre*, and control littermates (*Egfr^+/+^*, *Egfr^f/+^*, *Egfr^f/f^*, *Egfr^+/+^ Cyp19-Cre*, and *Egfr^f/+^ Cyp19-Cre*).

Immature (21–24 days old) female mice were injected i.p. with 5 IU PMSG to stimulate follicle development to the preovulatory stage. After 44 h, some animals were also injected i.p. with 5 IU hCG to induce differentiation of preovulatory follicles, oocyte maturation and ovulation. Ovaries were isolated at selected times after hormone priming and used as described herein and in the figure legends. The time course of in vivo GVBD was assessed by excising ovaries at specific times after hCG and placing them in L15 medium containing 5% fetal bovine serum (FBS), 100 U penicillin and 100 µg streptomycin. Preovulatory follicles were punctured to release the COCs, and oocytes were denuded of cumulus cells and evaluated for morphological evidence of GVBD under a stereomicroscope. For histological analyses, ovaries were fixed in Bouin's solution (Polysciences, Inc, Warrington, PA) overnight at 4°C, dehydrated and embedded in paraplast, sectioned serially at 5 µm onto Superfrost Plus slides (Fisher Scientific, Pittsburg, PA), stained with hematoxylin and eosin, and examined by light microscopy. For superovulation studies, oocytes were recovered from the oviducts ∼15–22 h post hCG and counted. To evaluate natural ovulation rates in adult female mice, mature females were mated with wild type males. On the morning a vaginal plug was observed, the female was euthanized and the number of oocytes in the oviducts was counted.

### Preovulatory follicle culture

Preovulatory follicles were microdissected from PMSG-primed mouse ovaries under a stereomicroscope, in Leibowitz's L15 medium supplemented with 5% FBS, 100 U penicillin and 100 µg streptomycin. Preovulatory follicles were next transferred through MEM supplemented with 10% FBS, 100 U penicillin and 100 µg streptomycin (MEM complete medium) three times, then cultured in 1 mL MEM complete medium under 95% O_2_/5% CO_2_. After equilibration, the preovulatory follicles (∼10–30 follicles/group) were cultured for 30 min in the absence or presence of AG1478 (0.5 µM), U0126 (20 µM), SB202190 (10 µM), or vehicle (DMSO) prior to stimulation with rLH (5 IU/mL) or AREG (100 nM). No differences in responses were observed when 10 or 30 follicles were used. At the end of culture, follicles were washed in DPBS and homogenized in ice-cold RIPA buffer containing a cocktail of EDTA-free protease inhibitors plus 1 mM EDTA, 1 mM Na-orthovanadate, 1 mM NaF and 1 mM Na-pyrophosphate to extract proteins. After centrifugation for 5 min at 4°C, supernatants were collected and protein concentrations assayed using the BCA Protein Assay Kit (Pierce, Rockford, IL). Additional preovulatory follicles from wild type, *Areg^−/−^ Egfr^wa2/^* ^wa2^ and *Egfr^delta/f^ Cyp19-Cre* mice were stimulated with 5 IU rLH for 0, 0.5, 1 and 2 h. Proteins were extracted in RIPA buffer as described above. The groups of wild type follicles used in the time course of rLH stimulation for wild type vs. *Areg^−/−^ Egfr^wa2/^* ^wa2^ were from either C57BL/6 mice or from *Areg^+/+^ Egfr^+/+^* and *Areg^+/+^ Egfr^wa2/+^* mice combined (C57BL/6J X 129Sv background). No differences in Western blotting results were observed between samples from mice on the C57BL/6 and the mixed backgrounds. Therefore, the wild type data generated using these samples were averaged. Littermate controls were used for wild type vs. *Egfr^delta/f^ Cyp19-Cre* follicle samples.

For the oocyte maturation studies, preovulatory follicles (∼10–12 follicles/group) were cultured as described above and stimulated as described in the figure legends. After 4 h, follicles were punctured using 26-1/2 gauge needles (Becton Dickinson & Co., Franklin Lakes, NJ) to release COCs. Cumulus cells were gently removed using a Drummond Microdispenser and oocytes were examined for evidence of GVBD.

### Western blotting

Preovulatory follicle protein samples were prepared as described above. Granulosa cells were isolated from PMSG-primed ovaries or from adult mouse ovaries by needle puncture in L15 medium, passed through a 40 µm nylon cell strainer (Becton Dickinson & Co.) and centrifuged at 1000 g, 4°C. After removal of the media, cells were washed once with PBS and centrifuged at 1000 g, 4°C. The PBS was removed, cells were homogenized in RIPA buffer as described above, and centrifuged for 5 min at 4°C. The supernatants were transferred to clean eppendorf tubes and protein concentrations were assayed using the Pierce BCA Protein Assay Kit.

Protein samples were separated on 7.5% or 10% polyacrylamide gels and transferred to PVDF membranes. Membranes were blocked for 1 h in TBST (Tris buffered saline + 0.1% Tween20) + 5% nonfat dry milk, washed in TBST, and incubated for 2 h at room temperature or overnight at 4°C with primary antibody diluted as follows: anti-phospho-AKT (1∶1000), anti-phospho-p38 MAPK (1∶1000), anti-phospho-Cx43(Ser262)(1∶200), and anti-phospho-Cx43(Ser255)(1∶200) in TBST + 5% BSA; anti-EGFR (1∶200) and anti-phospho-MAPK (1∶1000) in TBST + 0.2% nonfat dry milk. After incubation with primary antibody, membranes were washed in TBST and then incubated for 1 h at room temperature with anti-rabbit or anti-mouse IgG-HRP. Specific signals were detected using ECL or ECL Plus reagent and visualized by autoradiography.

Afterwards, membranes were stripped of bound antibodies by incubation for 30 min at 50°C in a solution containing 62.5 mM Tris-HCl pH 6.8, 2% SDS and 100 mM β-mercaptoethanol. Membranes were washed in TBST, blocked in TBST + 1% nonfat dry milk and then incubated with primary antibody for 2 h at room temperature or overnight at 4°C. Primary antibodies were diluted as follows: anti-AKT (1∶1000) and anti-p38 MAPK (1∶1000) in TBST + 5% BSA; anti-ERK1 (1∶2500) and anti-Cx43 (1∶1000) in TBST + 0.2% nonfat dry milk. After washing in TBST, membranes were incubated for 1 h at room temperature with anti-rabbit or anti-mouse IgG-HRP. Specific signals were detected using ECL or ECL Plus Western Blotting reagent and visualized by autoradiography. Bands were scanned and quantified by densitometric analyses using Adobe Photoshop CS3.

### Immunohistochemistry

Immunohistochemistry was performed as previously described [12].

### Measurement of cGMP levels

Preovulatory follicles were cultured as described above. Preovulatory follicles were preincubated with or without U0126 (20 µM) or AG1478 (0.5 µM) for 30 min, then stimulated with AREG (100 nM) or rLH (5 IU/mL) for 2 h. At the end of culture, the follicles were washed in PBS and then transferred to clean eppendorf tubes. The PBS was aspirated, 100 µl of 5% trichloroacetic acid (TCA) was added, and follicles were homogenized on ice using blue dounce homogenizers. Samples were frozen in liquid nitrogen and stored at −80°C until ready to proceed. After thawing, samples were homogenized again, on ice. The precipitate was removed by centrifugation and the supernatant was transferred to a clean eppendorf tube. The TCA was extracted from samples using water-saturated diethyl ether as described in the cGMP EIA kit. Samples were speed vacuumed to dryness and resuspended in 100 µl of EIA buffer. Samples and cGMP standards were acetylated, diluted, and quantified as described in the cGMP EIA kit.

### Statistics

Data are presented as the mean ± SEM or as mean ± range, as indicated in the figure legends. Data were analyzed by Student's t-test or two-way ANOVA where appropriate. P<0.05 was considered statistically significant.

## Results

### Disruption of *Egfr* in mouse ovarian granulosa cells

**Figure 1:**
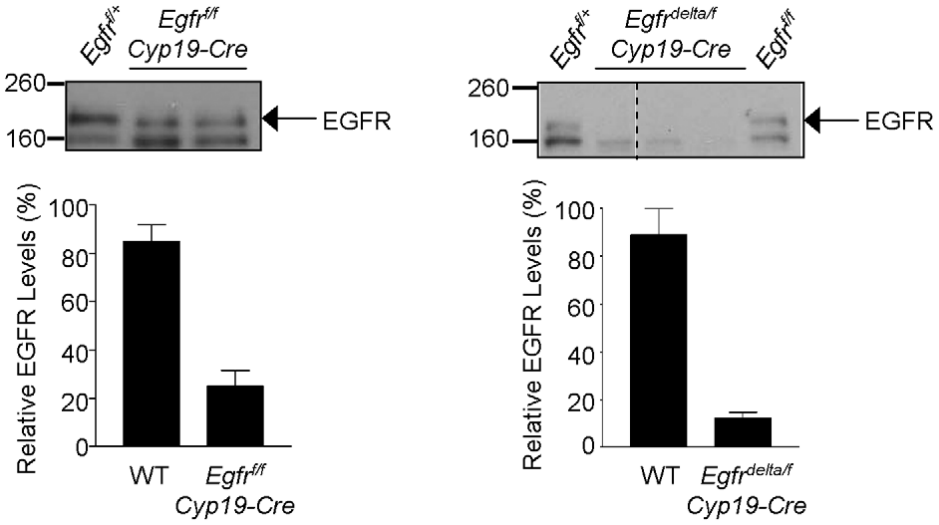
Conditional knockout of EGFR in granulosa cells from mouse preovulatory follicles. Western blot analysis was performed to detect EGFR protein (170 kDa) in granulosa cells isolated from preovulatory follicles of PMSG-primed wild type (WT), *Egfr^f/f^ Cyp19-Cre* and *Egfr^delta/f^ Cyp19-Cre* mice. Representative blots are shown. The densities for the WT, *Egfr^f/f^ Cyp19-Cre* and *Egfr^delta/f^ Cyp19-Cre* bands were normalized to one of the WT bands on the same blot, averaged and expressed as percentage.

In order to analyze the role of the EGFR in regulating LH-induced oocyte maturation and ovulation, we sought to generate mice with granulosa cell-specific knockout of the EGFR. Mice carrying a conditional allele of *Egfr* (*Egfr^f^*) [29] were crossed with *Cyp19-Cre* transgenic mice [30] to generate *Egfr^f/f^ Cyp19-Cre* mice; the gonad-specific *Cyp19* promoter expresses Cre specifically in the granulosa cells of growing follicles [30]. Cre-mediated recombination of the *Egfr^f^* allele was confirmed by PCR (not shown), and Western blot analysis was performed to detect EGFR protein (∼170 kDa) in granulosa cells from PMSG-primed wild type and *Egfr^f/f^ Cyp19-Cre* mouse ovaries. EGFR levels were reduced but not depleted in granulosa cells from *Egfr^f/f^ Cyp19-Cre* mouse ovaries (Figure 1). To further decrease EGFR expression in granulosa cells of ovarian follicles, mice carrying a null allele of *Egfr* (*Egfr^delta^*) were crossed with the *Egfr^f/f^ Cyp19-Cre* mice to generate *Egfr^delta/f^ Cyp19-Cre* mice. Western blot analysis revealed little to no EGFR protein in granulosa cells isolated from PMSG-primed *Egfr^delta/f^ Cyp19-Cre* mouse ovaries (Figure 1).

### Impaired oocyte meiotic resumption and cumulus expansion in *Egfr* conditional knockout mouse ovaries

**Figure 2:**
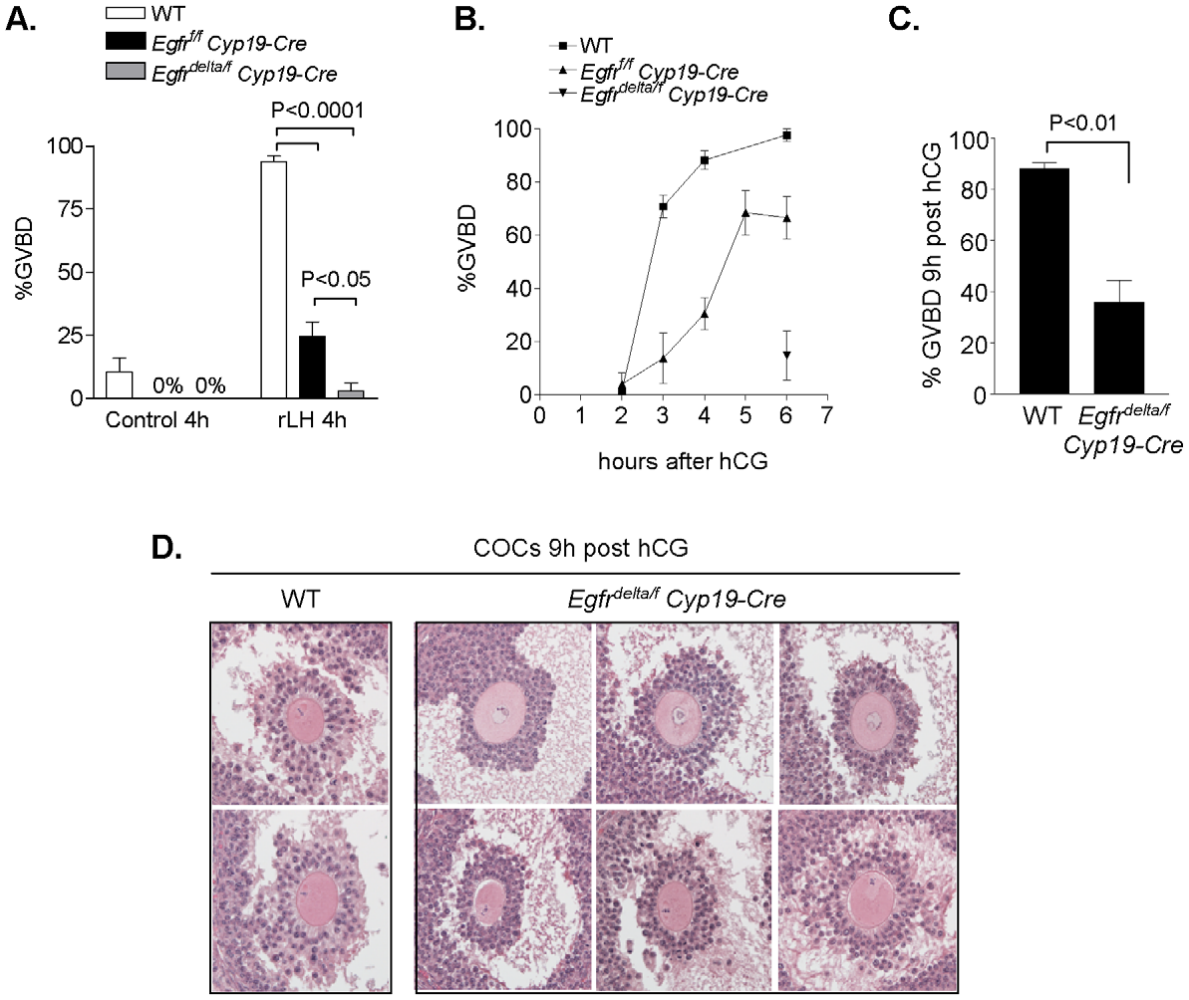
Impaired oocyte meiotic resumption and cumulus expansion in EGFR granulosa cell-specific knockout mouse preovulatory follicles. **A,** WT, *Egfr^f/f^ Cyp19-Cre* and *Egfr^delta/f^ Cyp19-Cre* preovulatory follicles were cultured in the presence or absence of rLH (5 IU) for 4 h. At the end of culture, oocytes were released from the follicles, denuded, and scored for GVBD. Data are the mean ± SEM of 3–12 samples for each treatment group. **B,** To evaluate the in vivo time course of oocyte maturation, oocytes were isolated from preovulatory follicles of mouse ovaries at the indicated times after hCG stimulation, denuded, and scored for GVBD. Data are the mean ± SEM for oocytes from 3–5 animals at each time point. **C,** Oocytes in preovulatory follicles were examined for evidence of GVBD through serial sections of WT (n = 4) and *Egfr^delta/f^ Cyp19-Cre* (n = 7) mouse ovaries that had been stimulated for 9 h with hCG. Data are the mean ± SEM. **D,** Representative images of hematoxylin- and eosin-stained COCs in preovulatory follicles of WT and *Egfr^delta/f^ Cyp19-Cre* mouse ovaries after 9 h hCG stimulation are shown.

The effect of EGFR depletion in granulosa cells of preovulatory follicles on oocyte meiotic resumption and cumulus expansion was next examined. Oocytes isolated from preovulatory follicles of PMSG-primed *Egfr^f/f^ Cyp19-Cre* and *Egfr^delta/f^ Cyp19-Cre* mouse ovaries underwent spontaneous maturation at a similar rate as wild type oocytes in vitro (data not shown), indicating that these oocytes had acquired meiotic competence. However, when preovulatory follicles were cultured in the presence of recombinant LH (rLH) for 4 h, oocyte meiotic resumption, scored as percent germinal vesicle breakdown (GVBD), was significantly impaired in both *Egfr^f/f^ Cyp19-Cre* and *Egfr^delta/f^ Cyp19-Cre* preovulatory follicles, compared to wild type (Figure 2A). When oocyte maturation was evaluated *in vivo*, oocyte meiotic resumption was found to occur with a delayed time course in *Egfr^f/f^ Cyp19-Cre* mice. At 5–6 h after hCG stimulation, ∼66-68% of oocytes scored had undergone GVBD (Figure 2B). In contrast, only ∼15% of *Egfr^delta/f^ Cyp19-Cre* oocytes had resumed meiosis at 6 h post hCG. After 9 h hCG, oocyte reentry into the cell cycle was still significantly impaired in *Egfr^delta/f^ Cyp19-Cre* ovaries (Figure 2C). In addition, oocytes were surrounded by compact or minimally expanded cumulus cell layers at this same time in *Egfr^delta/f^ Cyp19-Cre* preovulatory follicles (Figure 2D), consistent with the phenotype observed in the *Areg^−/−^ Egfr^wa2/wa2^* mouse model [8].

### Impaired ovulation and reduced fecundity in *Egfr* conditional knockout mice

**Figure 3:**
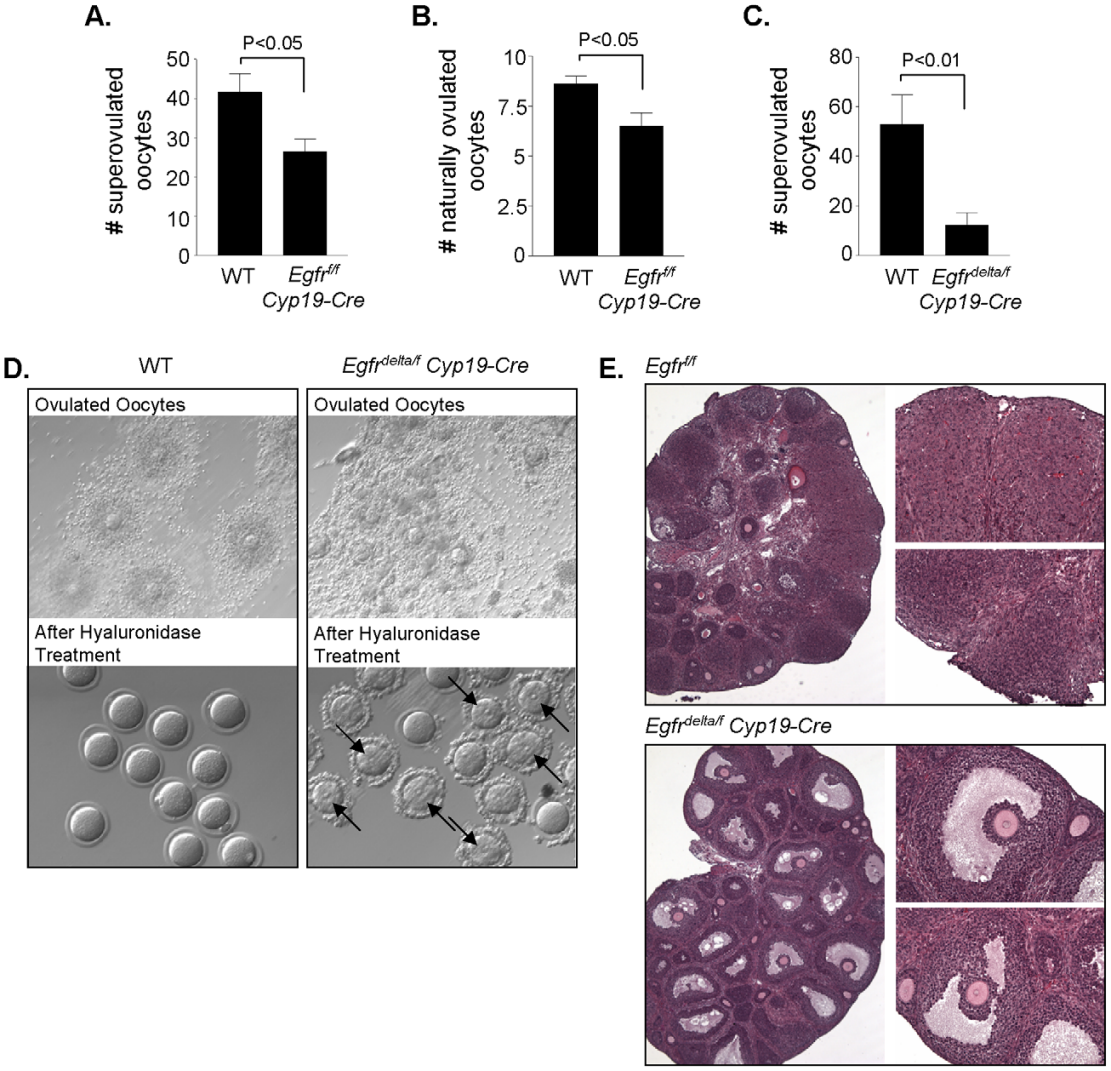
Ovulation is impaired in *Egfr^f/f^ Cyp19-Cre* and *Egfr^delta/f^ Cyp19-Cre* mice. **A and C,** When super-ovulated, *Egfr^f/f^ Cyp19-Cre* and *Egfr^delta/f^ Cyp19-Cre* mice released significantly fewer oocytes into the oviducts than WT females. The greater the disruption of *Egfr* expression in mice, the fewer the number of oocytes ovulated. Data are the mean ± SEM (n = 15–16 for WT vs. *Egfr^f/f^ Cyp19-Cre*, and n = 4–6 for WT vs. *Egfr^delta/f^ Cyp19-Cre*). **B,** Adult WT and *Egfr^f/f^ Cyp19-Cre* females were mated with WT males. On the morning a vaginal plug was observed, the females were euthanized and the number of naturally ovulated oocytes retrieved from the oviducts were counted. Data are the mean ± SEM (WT, n = 5; *Egfr^f/f^ Cyp19-Cre*, n = 4). **D**, Representative images show the morphology of super-ovulated COCs from the oviducts of WT and *Egfr^delta/f^ Cyp19-Cre* females. The cumulus cells surrounding *Egfr^delta/f^ Cyp19-Cre* oocytes appear disorganized compared to the WT. WT oocytes were easily denuded with hyaluronidase treatment, whereas many oocytes from *Egfr^delta/f^ Cyp19-Cre* mice were still surrounded by compact cumulus cells after hyaluronidase treatment. Arrows point to oocytes that were still in GV after removal of the compact cumulus cells. **E,** Histology of super-ovulated WT (*Egfr^f/f^*) and *Egfr^delta/f^ Cyp19-Cre* mouse ovaries. Newly formed corpora lutea are observed in the WT ovary, whereas large antral follicles with entrapped GV oocytes surrounded by compact cumulus cells are present in the *Egfr^delta/f^ Cyp19-Cre* ovary.

To determine the effect of disruption of *Egfr* in granulosa cells on ovulation, *Egfr^f/f^ Cyp19-Cre*, *Egfr^delta/f^ Cyp19-Cre*, and control littermate females were stimulated with PMSG and hCG as described in the Materials and Methods. At 15–22 h post hCG, the ovulated COCs were released from the oviducts and counted. *Egfr^f/f^ Cyp19-Cre* females super-ovulated an average of 26.56±3.15 oocytes, which was significantly less than the 41.65±4.73 oocytes ovulated by littermate controls (Figure 3A). To determine the natural ovulation rates for adult females, females were mated with wild type males and euthanized on the morning a vaginal plug was observed. Adult *Egfr^f/f^ Cyp19-Cre* females ovulated an average of 6.50±0.74 oocytes into the oviducts, compared to 8.6±0.45 oocytes in wild type adult female littermates (P<0.05)(Figure 3B). Thus, partial knockdown of EGFR in granulosa cells results in a small but significant decrease in natural ovulation of oocytes.

Impaired super-ovulation was more profound in *Egfr^delta/f^ Cyp19-Cre* females which ovulated only 12±5.5 oocytes, compared to 53±13.72 oocytes in littermate controls (Figure 3C). Interestingly, the cumulus cells surrounding the super-ovulated oocytes from both *Egfr^f/f^ Cyp19-Cre* (not shown) and *Egfr^delta/f^ Cyp19-Cre* (Figure 3D) mice were observed to be poorly organized; although there was some expansion of the outer cumulus cell layers, these cells were in clumps, in contrast to the uniformly expanded cumulus cells of ovulated wild type COCs (Figure 3D). In addition, the inner cumulus cell layers surrounding many of the ovulated oocytes from *Egfr^f/f^ Cyp19-Cre* and *Egfr^delta/f^ Cyp19-Cre* mice were compact and could not be removed with hyaluronidase treatment (Figure 3D). When oocytes from the latter mice were denuded of the compact cumulus cells, many were still in the GV stage (average 44.87±10.15% of total superovulated oocytes, n = 4). However, once denuded, these oocytes underwent spontaneous maturation. Ovulated oocytes that were not surrounded by compact layers of cumulus cells had already undergone meiotic resumption.

**Figure 4:**
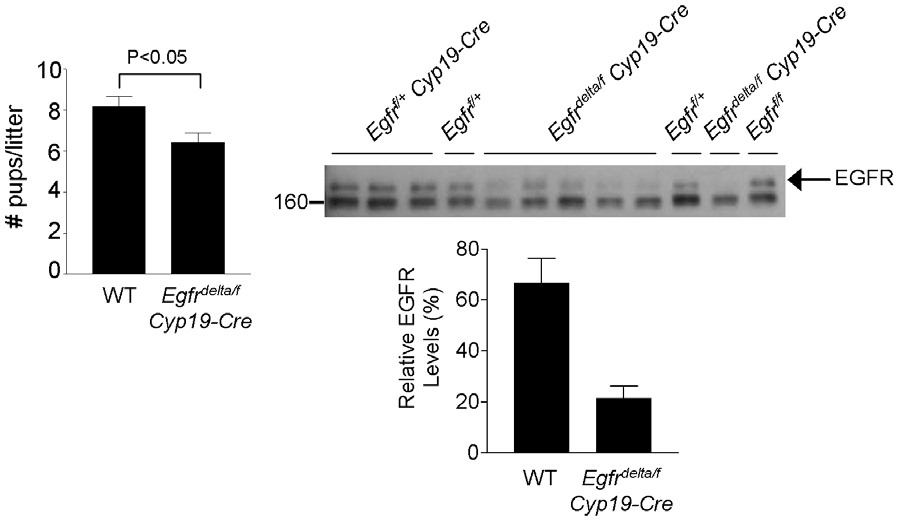
Reduced fecundity of *Egfr^delta/f^ Cyp19-Cre* females due to incomplete disruption of EGFR in granulosa cells. Adult WT and *Egfr^delta/f^ Cyp19-Cre* females were mated at 6 weeks of age with WT males and the number of pups born per litter was recorded over a period of 4–6 months (n = 5 for each genotype). Western blot analysis was performed to detect EGFR protein (170 kDa) in granulosa cells isolated from adult female mice with the indicated genotypes. The densities for the WT and *Egfr^delta/f^ Cyp19-Cre* EGFR bands were normalized to one of the WT bands on the same blot, averaged and expressed as percentage.

Because *Egfr^delta/f^ Cyp19-Cre* mice released few oocytes into the oviducts, the histology of the super-ovulated ovaries was examined. Large antral follicles containing GV-stage oocytes surrounded by compact cumulus cells were observed (Figure 3E), whereas luteinizing structures were present in the super-ovulated ovaries of wild type controls. Surprisingly, when adult *Egfr^delta/f^ Cyp19-Cre* females were mated with wild type males over a period of 4–6 months, they produced an average of 6.43±0.49 pups/litter, compared to 8.17±0.51 pups/litter for wild type females (P<0.05)(Figure 4). Western blot analyses revealed reduced but detectable levels of EGFR protein in granulosa cells isolated from adult *Egfr^delta/f^ Cyp19-Cre* ovaries compared to wild type (Figure 4). Thus, although little to no EGFR protein could be detected in granulosa cells from hormone-primed immature *Egfr^delta/f^ Cyp19-Cre* females, knockdown of EGFR appeared less efficient during the natural cycle of adult mutant females. A possible explanation is that the exogenous PMSG stimulus activates CRE expression more effectively than the endogenous FSH increase.

### LH-induced phosphorylation of p38MAPK and MAPK3/1 but not AKT is dependent in part on signaling through a functionally active EGFR

**Figure 5:**
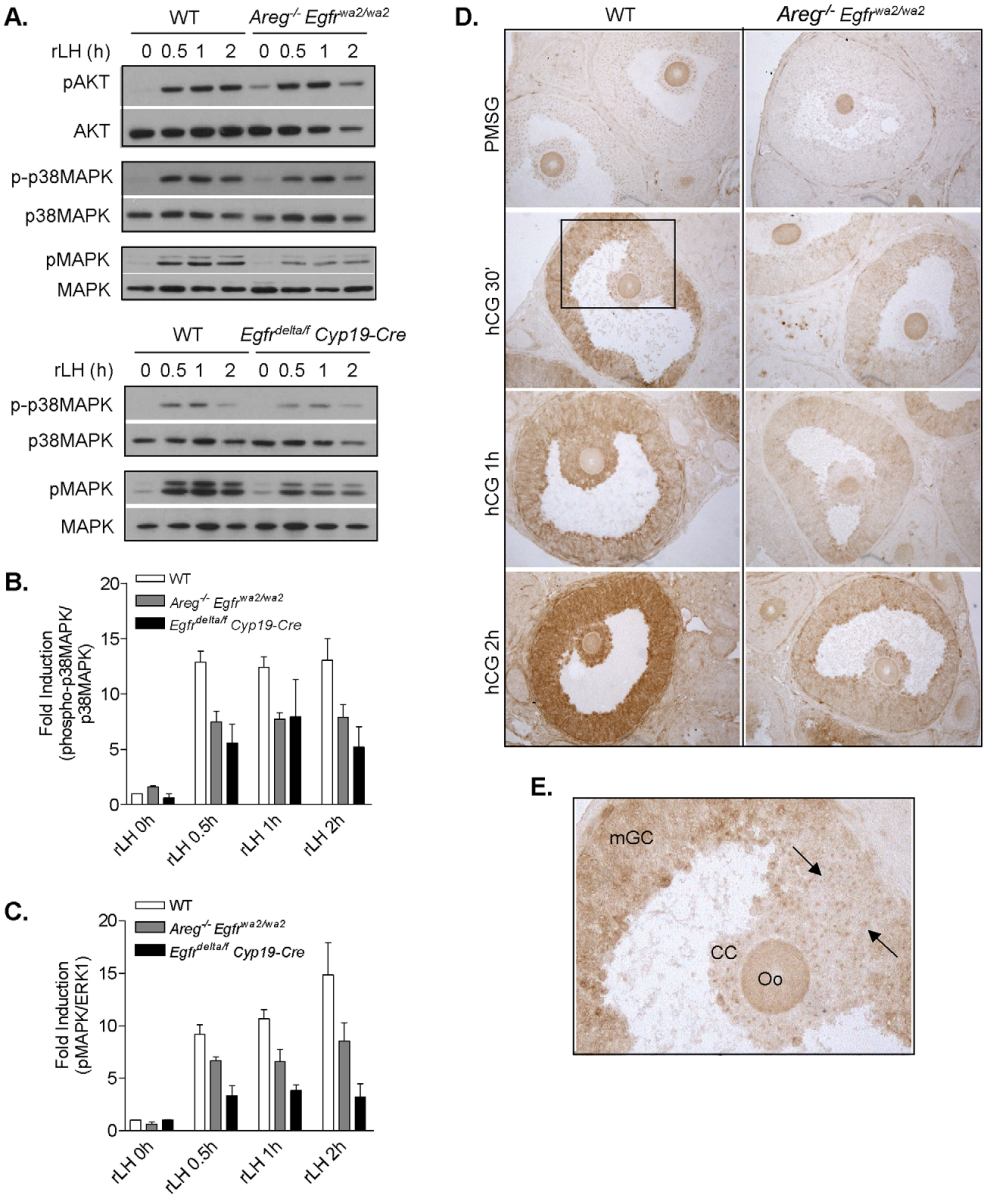
LH-induced phosphorylation of p38MAPK and MAPK3/1, but not AKT, is dependent on EGFR signaling. Western blot analysis was performed to compare the time course of rLH (5 IU)-induced AKT phosphorylation, p38MAPK phosphorylation, and MAPK3/1 phosphorylation in WT, *Areg^−/−^ Egfr^wa2/wa2^* and *Egfr^delta/f^ Cyp19-Cre* preovulatory follicles. **A,** Representative blots are shown. **B,** The ratio of phosphorylated p38MAPK to p38MAPK was normalized to WT control (0 h) and expressed as fold induction. Data are the mean ± SEM of at least three separate experiments. For WT vs. *Areg^−/−^ Egfr^wa2/wa2^* and WT vs. *Egfr^delta/f^ Cyp19-Cre*, P<0.001 and P<0.0001, respectively, by two-way ANOVA. **C,** The ratio of phosphorylated MAPK3/1 to MAPK3/1 was normalized to WT control (0 h) and expressed as fold induction. Data are the mean ± SEM of at least three separate experiments, except for *Egfr^delta/f^ Cyp19-Cre* samples for MAPK3/1 analysis (n = 2, range reported). P<0.01 for WT vs. *Areg^−/−^ Egfr^wa2/wa2^* and P<0.001 for WT vs. *Egfr^delta/f^ Cyp19-Cre* by two-way ANOVA. **D,** Phosphorylated MAPK3/1 was localized in PMSG- and hCG-primed ovaries of WT and *Areg^−/−^ Egfr^wa2/wa2^* mice by immunohistochemistry using an anti-phospho-p44/42 MAPK antibody. **E,** Closer view of phospho-MAPK3/1 immunolocalization in the wild type follicle after 30 min hCG (boxed area in D). Activated MAPK3/1 is present in the mural granulosa cells (mGC) lining the antrum, but is absent/low in the cumulus cells (CC) surrounding the oocyte (Oo) and in the granulosa cells adjacent to the COC (the cells between the arrows). Staining of the oocytes was not observed in all experiments and was considered non-specific.

To elucidate downstream targets of EGFR that may be important for oocyte meiotic resumption, we examined the activation of different signaling pathways downstream of LH and EGFR using two different genetic models of disruption of the EGF network (the *Areg^−/−^ Egfr^wa2/wa2^* and *Egfr^delta/f^ Cyp19-Cre* mutant mouse models) and the preovulatory follicle culture model. Preovulatory follicles isolated from PMSG-primed wild type and mutant ovaries were cultured with or without rLH for 0, 0.5, 1 and 2 h. In *Areg^−/−^ Egfr^wa2/wa2^* preovulatory follicles, rLH induced AKT phosphorylation within 30 min to similar levels as those observed in wild type follicles (Figure 5A). Phosphorylated AKT levels remained elevated and were not significantly different between wild type and *Areg^−/−^ Egfr^wa2/wa2^* preovulatory follicles after up to 2 h of rLH stimulation (Figure 5A and analysis not shown). This finding is consistent with those by Andric et al. [31], who examined hCG-induced phosphorylation of AKT in whole ovary lysates of *wa2* and *velvet* mice. Therefore, AKT phosphorylation was not examined further in *Egfr^delta/f^ Cyp19-Cre* follicles. Taken together, LH activation of AKT does not appear dependent on EGFR signaling.

In wild type preovulatory follicles, rLH stimulated p38MAPK phosphorylation within 30 min and the phosphorylation levels remained elevated up to 2 h after rLH (Figure 5A and 5B). Phosphorylation of p38MAPK was also induced by LH in *Areg^−/−^ Egfr^wa2/wa2^* follicles within 30 min, but to lower levels (Figure 5A and 5B). The amount of phospho-p38MAPK remained lower in the *Areg^−/−^ Egfr^wa2/wa2^* versus wild type follicles up to 2 h post rLH. Similarly, lower levels of phosphorylated p38MAPK were induced by LH in preovulatory follicles of *Egfr^delta/f^ Cyp19-Cre* mice compared to wild type (Figure 5A and 5B). Taken together, LH-induced p38MAPK activation is regulated by EGFR.

As previously reported [12], rLH-induced phosphorylation of MAPK3/1 in wild type preovulatory follicles occurs within 30 min of stimulation, and is elevated up to 2 h post rLH (Figure 5A and 5C). In both the *Areg^−/−^ Egfr^wa2/wa2^* and *Egfr^delta/f^ Cyp19-Cre* follicles, rLH also induced MAPK3/1 phosphorylation within 30 min, to levels that were lower than that in the wild type follicles. Consistent with our previous results [12], MAPK3/1 phosphorylation was significantly reduced but not completely prevented in both *Areg^−/−^ Egfr^wa2/wa2^* and *Egfr^delta/f^ Cyp19-Cre* follicles at 2 h after rLH, compared to the wild type (Figure 5C).

### Localization of Phosphorylated MAPK3/1 in Wild Type and *Areg^−/−^ Egfr^wa2/wa2^* Ovaries

Immunohistochemistry was performed to localize phosphorylated MAPK3/1 in PMSG- and hCG-primed ovaries of *Areg^−/−^ Egfr^wa2/wa2^* versus wild type mice. In both the wild type and *Areg^−/−^ Egfr^wa2/wa2^* ovaries, immune signal for phosphorylated MAPK3/1 was detected in the mural granulosa cells lining the antrum of preovulatory follicles, but not in cumulus cells after 30 min hCG (Figure 5D). In many of the follicles examined at this time point, activated MAPK3/1 in the granulosa cells adjacent to the COC also was absent/very low (Figure 5E). At 1 h hCG, phosphorylated MAPK3/1 localized to both the mural granulosa and cumulus cells of wild type preovulatory follicles, whereas the signal was observed primarily in the mural granulosa cells of *Areg^−/−^ Egfr^wa2/wa2^* preovulatory follicles (Figure 5D). The intensity of the immunostaining appeared strongest in mural granulosa and cumulus cells of wild type preovulatory follicles at 2 h hCG. Immunostaining was also detected in the cumulus cells and mural granulosa cells of *Areg^−/−^ Egfr^wa2/wa2^* preovulatory follicles at the same time. Taken together, the spatial and temporal pattern of MAPK3/1 activation in preovulatory follicles suggests that there is a diffusion of signal from the mural granulosa cells inward to the COC that is partially disrupted in follicles with impaired signaling through the EGF network. The delayed activation of MAPK3/1 in the granulosa cells directly adjacent to the COC also suggests that these cells differ from the mural granulosa cells lining the antrum due to their proximity to the COC.

### LH-induced connexin-43 phosphorylation is impaired in mouse preovulatory follicles with disruption of EGFR signaling

**Figure 6:**
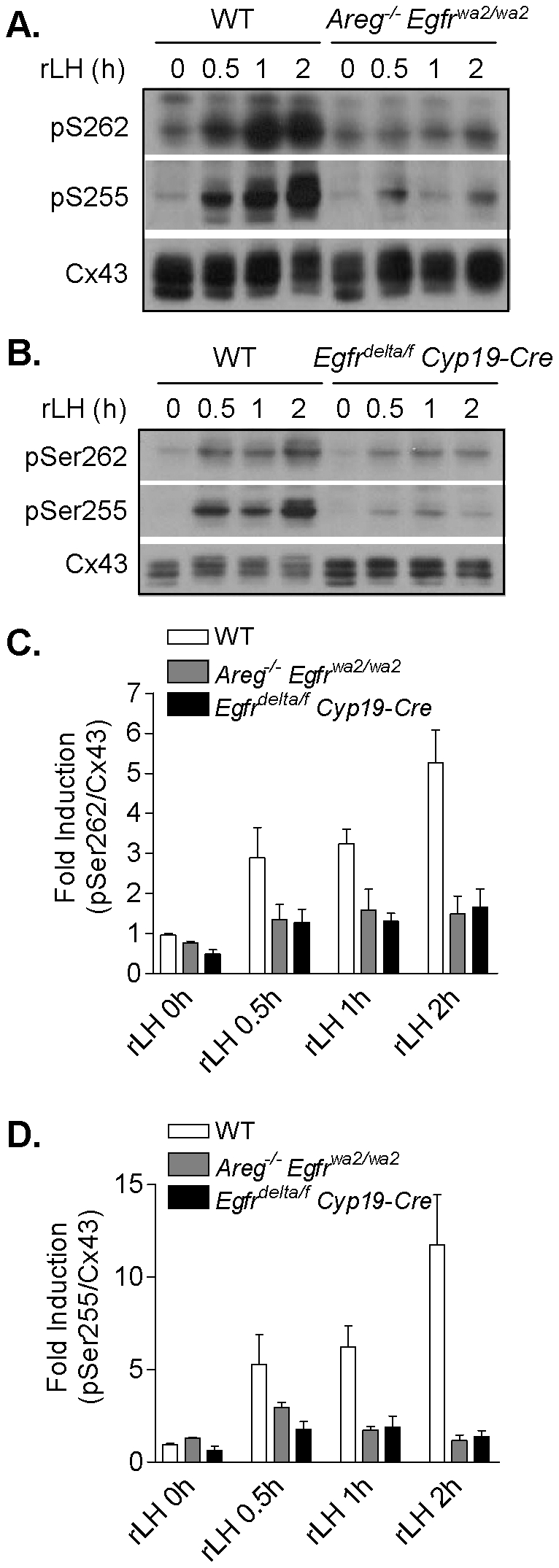
LH-induced connexin-43 phosphorylation is impaired in *Areg^−/−^ Egfr^wa2/wa2^* and *Egfr^delta/f^ Cyp19-Cre* mouse preovulatory follicles. The time course of rLH-induced Cx43 phosphorylation on S262 and S255 was examined in WT, *Areg^−/−^ Egfr^wa2/wa2^* and *Egfr^delta/f^ Cyp19-Cre* mouse preovulatory follicles by Western blot analyses. **A and B**, Representative blots are shown. **C and D**, The ratio of phosphorylated S262 or S255 to total Cx43 was normalized to the wild type control and expressed as fold induction. Data are the mean ± SEM of at least three separate experiments and were analyzed by two-way ANOVA. For pSer262/Cx43, P<0.001 for both WT vs. *Areg^−/−^ Egfr^wa2/wa2^* and WT vs. *Egfr^delta/f^ Cyp19-Cre*. For pSer255/Cx43, P<0.01 for both WT vs. *Areg^−/−^ Egfr^wa2/wa2^* and WT vs. *Egfr^delta/f^ Cyp19-Cre*.

LH has been shown to cause MAPK3/1-dependent phosphorylation and closure of Cx43 gap junctions prior to oocyte meiotic resumption in mouse preovulatory follicles [26]. Here, we examined the dependence of phosphorylation of Cx43 on S262 and 255, two of the serine residues implicated in gap junction closure[26], on EGFR. Wild type, *Areg^−/−^ Egfr^wa2/wa2^*, and *Egfr^delta/f^ Cyp19-Cre* preovulatory follicles were cultured in the presence of rLH for 0, 0.5, 1 and 2 h. In this follicle culture model, rLH stimulation of preovulatory follicles from both mutant mouse lines produced no oocyte maturation at 4 h (Figure 2A and data not shown). In wild type follicles, rLH induced Cx43 phosphorylation on both S262 and S255 within 30 min, and phosphorylation of these two serine residues was further increased between 1 and 2 h after rLH (Figure 6). In the *Areg^−/−^ Egfr^wa2/wa2^* and *Egfr^delta/f^ Cyp19-Cre* follicles, rLH induced a small increase in Cx43 phosphorylation on S262 and S255 within 30 min, to levels that were lower than but not significantly different from the wild type (Figure 6). However, rLH failed to further increase Cx43 phosphorylation on either serine residues in the mutant follicles at 1 and 2 h after stimulation (Figure 6). Thus, LH-induced Cx43 gap junction phosphorylation is dependent on EGFR signaling.

### LH-induced connexin-43 phosphorylation in preovulatory follicles is prevented by the EGFR inhibitor AG1478 and the p38MAPK inhibitor SB202190

**Figure 7:**
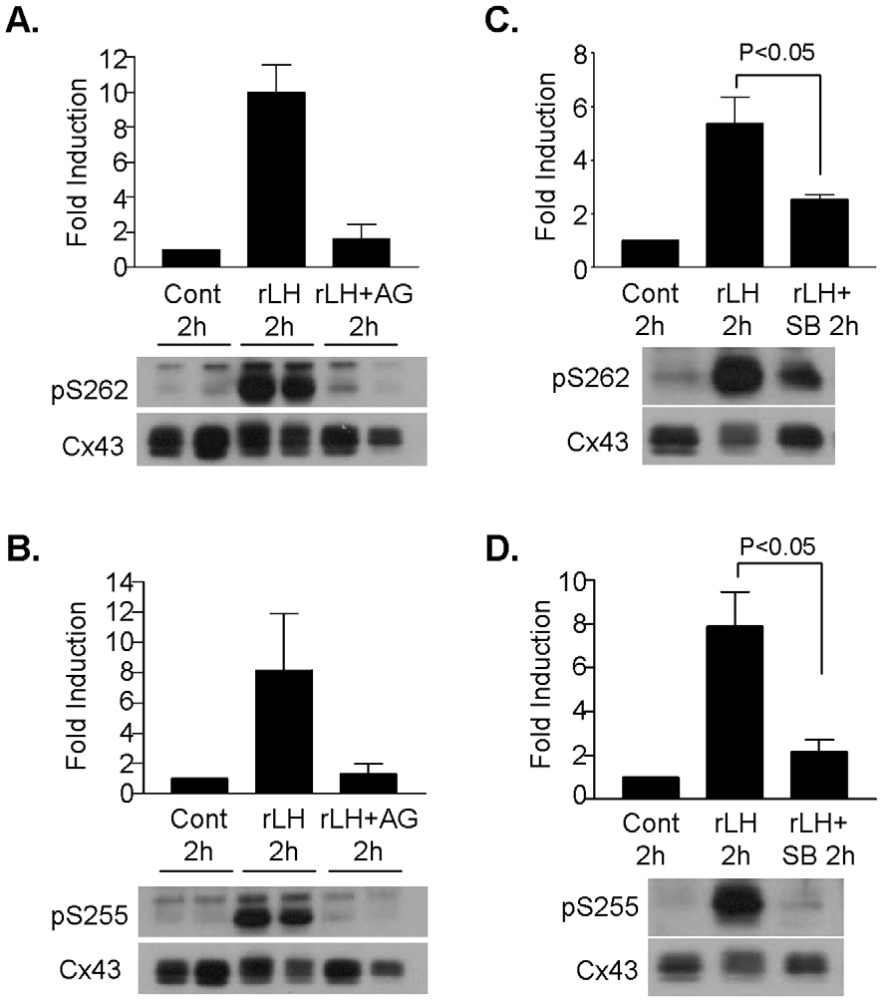
Inhibition of LH-induced connexin-43 phosphorylation by the EGFR inhibitor AG1478 and the p38MAPK inhibitor SB202190. **A and B,** LH-induced Cx43 phosphorylation on S262 and S255 was examined by Western blot analyses using protein extracts from preovulatory follicles that were pre-incubated for 30 min with or without the EGFR tyrosine kinase inhibitor AG1478 (0.5 µM), and then stimulated for 2 h with rLH (5 IU). The ratio of phosphorylated S262 or S255 to total Cx43 was normalized to the control and expressed as fold induction. Data are the mean ± range of two separate experiments, and are shown in the immunoblots. **C and D,** Western blot analyses to detect LH-induced phosphorylation of Cx43 on S262 and S255 was performed using protein extracts from preovulatory follicles pre-incubated for 30 min with or without SB202190 (10 µM) and stimulated for 2 h with rLH (5 IU). The ratio of phosphorylated S262 or S255 to total Cx43 was normalized to the control and expressed as fold induction. Data are the mean ± SEM of three separate experiments. Representative blots are shown.

To further document the dependence of LH-induced Cx43 phosphorylation on EGFR tyrosine kinase activity, wild type preovulatory follicles were incubated for 30 min with or without the EGFR tyrosine kinase inhibitor AG1478 prior to stimulation with rLH for 2 h. Consistent with what was observed in the mutant follicles, AG1478 completely blocked LH-induced phosphorylation of Cx43 on both S262 and S255 (Figures 7A and 7B).

LH-induced phosphorylation and closure of Cx43 gap junctions has been shown to be dependent on MAPK3/1 [26]. Here, we examined whether inhibition of p38MAPK activation also affected LH-induced Cx43 phosphorylation. Pretreatment of follicles with SB202190 inhibited rLH-induced phosphorylation of Cx43 on S262 and S255 by ∼55% and ∼66%, respectively (Figure 7C and 7D). Thus, rLH-stimulated Cx43 phosphorylation is dependent not only on the MAPK3/1 pathway, but also in part on activation of the p38MAPK pathway. These results also suggest that phosphorylation of S255 is more sensitive to activated p38MAPK than S262. An effect of SB202190 on oocyte maturation was also observed. When preovulatory follicles were preincubated with 10 µM SB202190 for 30 minutes, rLH stimulated only 43.43±11.1% GVBD after 4 hours, compared to 85.5±3.15% GVBD with rLH alone (P<0.01).

### The MEK1 inhibitor U0126 partially inhibits AREG-induced oocyte GVBD but completely blocks AREG-induced connexin-43 phosphorylation

**Figure 8:**
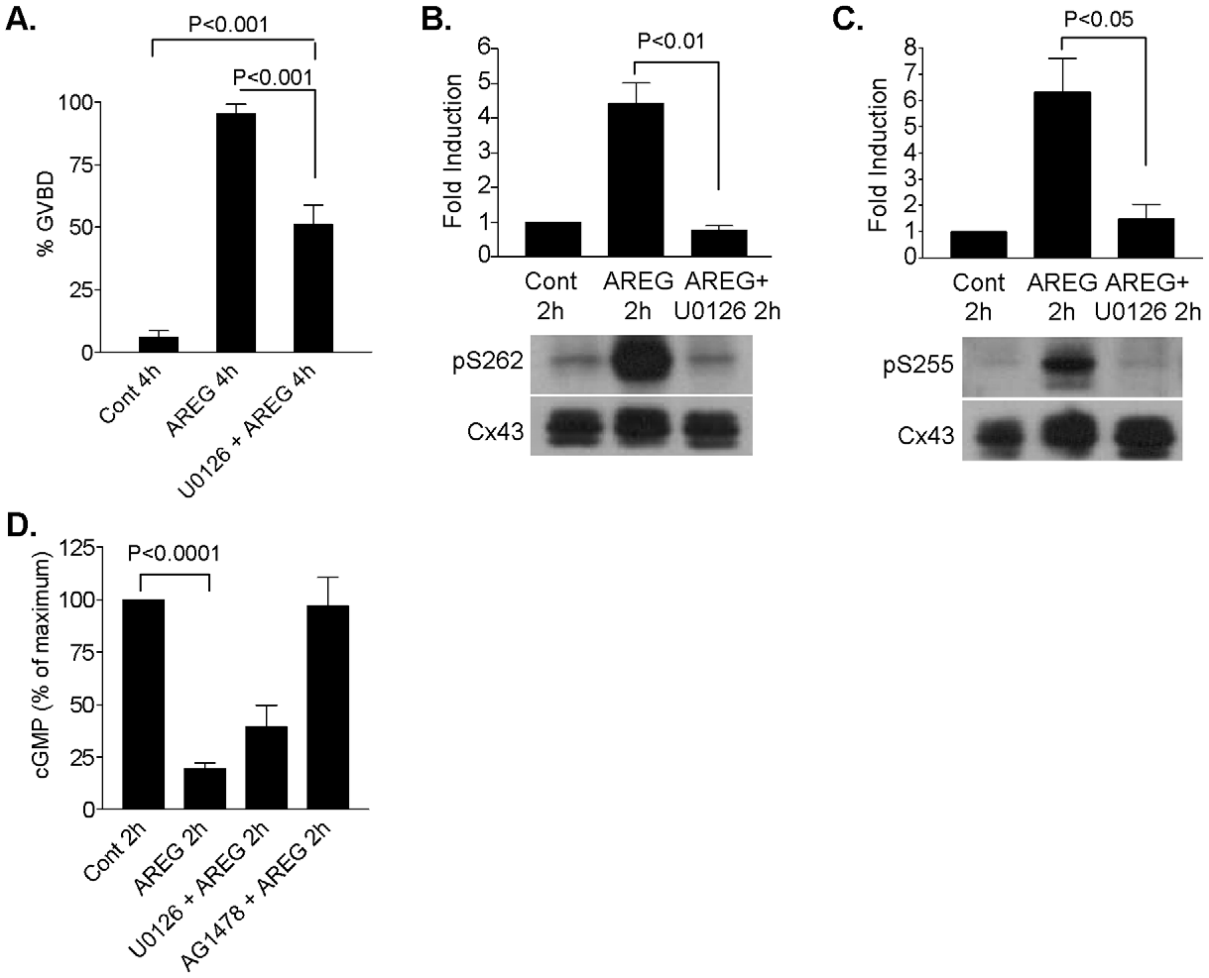
Effect of U0126 on AREG-induced connexin-43 phosphorylation, oocyte maturation, and cGMP levels. **A,** WT preovulatory follicles were preincubated for 30 min with or without U0126 (20 µM), and then stimulated with AREG (100 nM) for 4 h. Oocytes were then released from the follicles and denuded of cumulus cells. The percentage of oocytes that underwent GVBD was scored. Data are the mean ± SEM of eight separate samples for each treatment group. **B and C,** Preovulatory follicles were preincubated for 30 min in the presence or absence of U0126 (20 µM), and then stimulated with AREG (100 nM) for 2 h. Protein extracts were prepared and used in Western blot analyses to detect the phosphorylation of Cx43 on S262 and S255. The ratio of phosphorylated S262 or S255 to total Cx43 was normalized to control. Data are the mean ± SEM of three separate experiments. Representative blots are shown. **D,** Cyclic GMP levels were measured in preovulatory follicles cultured with or without U0126 (20 µM) or AG1478 (0.5 µM) for 30 min followed by 2 h stimulation with AREG (100 nM). Data shown are the mean ± SEM of three separate experiments.

Recently, AREG was shown to induce a decrease in follicular cGMP content [22]. Decreased cGMP levels in the follicle may lead to relief of inhibition of PDE3A activity, resulting in decreased cAMP in the oocyte and meiotic resumption. Here, we examined the effect of the MEK inhibitor, U0126, on AREG-induced GVBD, Cx43 phosphorylation and decrease in cGMP in the follicle. In cultured preovulatory follicles, AREG-induced GVBD was significantly but incompletely inhibited by U0126 (Figure 8A). When preovulatory follicles were cultured in the presence of AREG for 2 h, phosphorylation of Cx43 on S262 and S255 was strongly induced (Figure 8B and 8C), whereas pretreatment with U0126 completely prevented AREG-induced phosphorylation of Cx43 on these serine residues (Figure 8B and 8C). As previously reported [22], AREG induced a significant decrease in cGMP in the follicle (Figure 8D). When preovulatory follicles were preincubated for 30 min with U0126, AREG still induced a decrease in cGMP, to levels that were not statistically different from that induced by AREG alone. Preincubation of follicles with AG1478 prevented the AREG-induced decrease in cGMP, indicating that the AREG effects are EGFR-dependent. This latter treatment completely blocks oocyte maturation (data not shown). Taken together, the data suggest that another pathway(s) in addition to the MAPK3/1 pathway is likely involved in promoting oocyte meiotic resumption.

### LH induces a decrease in cGMP in *Egfr^delta/f^ Cyp19-Cre* preovulatory follicles

**Figure 9:**
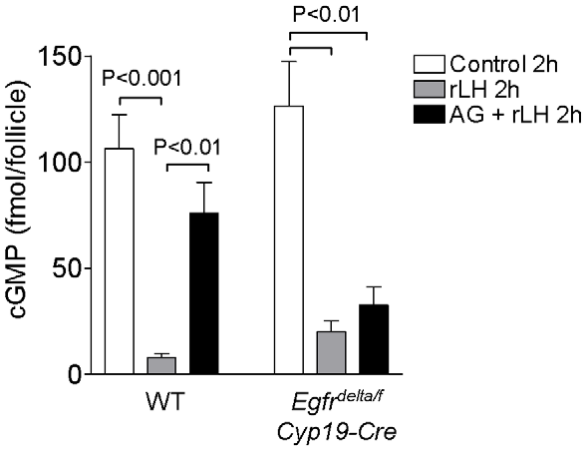
Comparison of cGMP levels in wild type and *Egfr^delta/f^ Cyp19-Cre* preovulatory follicles. WT and *Egfr^delta/f^ Cyp19-Cre* preovulatory follicles were preincubated with or without AG1478 (0.5 µM) for 30 min, and then stimulated with or without rLH (5 IU/mL) for 2 h. Cyclic GMP levels were measured as described in the Materials and Methods. Data are shown as the mean ± SEM of three or four separate samples for each treatment group.

The LH-induced decrease in cGMP in the preovulatory follicle is thought to trigger PDE3A activation in the oocyte, leading to decreased cAMP and oocyte meiotic resumption [21], [22]. Here, we investigated whether the failure of oocytes to reenter meiosis in *Egfr^delta/f^ Cyp19-Cre* preovulatory follicles might be due to disruption of the LH-induced decrease in intra-follicular cGMP. Levels of cGMP were measured in wild type and mutant follicles stimulated with or without rLH for 2 h. In unstimulated follicles from both wild type and mutant mice, cGMP levels were high (Figure 9). The cGMP levels decreased significantly in response to rLH stimulation of wild type preovulatory follicles. LH also induced a decrease in cGMP in *Egfr^delta/f^ Cyp19-Cre* follicles, but to levels that were approximately two-fold higher than that in the wild type follicles. The LH effects were prevented by the EGFR inhibitor AG1478 in wild type follicles, but not in follicles in which EGFR was absent, confirming the specificity of AG1478.

## Discussion

LH activation of its cognate receptor at the surface of granulosa and theca cells leads to activation of adenylyl cyclase and production of the second messenger, cAMP [32], [33]. LHR coupling to Gq/11 and Ca^2+^ signaling has also been reported (5). The LH signal then branches into activation of a myriad of intracellular signaling pathways that culminate in oocyte maturation, cumulus expansion and ovulation [34]. Recent evidence of LH-mediated transactivation of the EGF network adds to the complex set of molecular events thought to be necessary for ovulation. Using a genetic approach and mouse preovulatory follicle culture as the experimental model, our findings conclusively show that the EGFR and signals emanating from this receptor are essential for normal LH induction of oocyte meiotic resumption and ovulation. Further, the spatial propagation of the LH signal is dependent on the EGF network. We also provide evidence that multiple redundant signals converge to trigger oocyte maturation. Thus, all these findings lend further support to the hypothesis that the EGF network is an essential signaling component for ovulation of a mature egg.

Previous reports from our laboratory and another have used a hypomorphic allele of *Egfr* to probe its function during ovulation [8], [12], [31]. However, this strategy suffers from global suppression of EGFR signaling. To conclusively identify the role of the EGFR in LH-induced oocyte maturation and ovulation, we disrupted *Egfr* expression specifically in granulosa cells in mice. Graded EGFR inactivation in granulosa cells disrupts LH signaling and oocyte maturation. The time course of oocyte maturation in vivo is compromised, with a progressive delay in GVBD. Cumulus expansion is also impaired and associated with significantly decreased ovulation rates. Interestingly, several of the ovulated oocytes were surrounded by compact inner cumulus cell layers and were still in GV. Oocytes denuded of these cumulus cells underwent spontaneous maturation, ruling out the possibility that failure to mature was due to incompetence to reenter the cell cycle. This observation is surprising given the well-established concept that removal of oocytes surrounded by cumulus cells from the follicle environment triggers spontaneous maturation. A possible explanation is that upon disruption of EGFR signaling, the compacted cumulus cells continue to send a signal that maintains oocyte meiotic arrest. Alternatively, it is possible that a positive signal dependent on EGFR activation has not occurred. Only total cGMP content in the follicle was measured and found to be significantly decreased in the *Egfr^delta/f^ Cyp19-Cre* follicles. Thus, we cannot exclude the possibility that this nucleotide remains in the oocyte at levels sufficient to inhibit PDE3A. Indeed the phenotype of ovulated GV oocytes is similar to that of PDE3A KO mice [19]; therefore, PDE3A may not have been sufficiently activated, thus the persistent meiotic arrest. A difference between the *Egfr^delta/f^ Cyp19-Cre* and the *Pde3a^−/−^* phenotypes is that oocytes from the former resume meiosis when denuded, whereas they do not in the latter. This observation strongly suggests that the defect is not intrinsic to the oocyte and that a signal from the somatic cells maintains the meiotic arrest in the ovulated oocytes. Further experiments are required to define whether cGMP is the somatic signal responsible for this phenotype.

**Figure 10:**
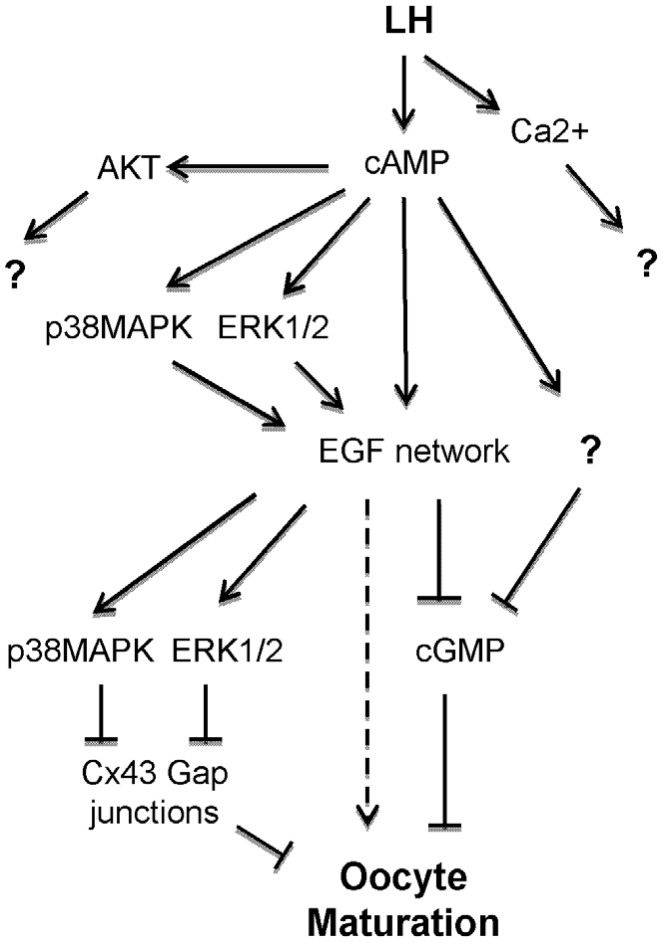
Summary of pathways involved in LH-induced EGFR transactivation and downstream signals involved in oocyte maturation. The broken arrow indicates possible stimulatory pathways that signal maturation. The consequences of Ca^2+^ signaling are presently unknown. Although AKT is involved in cell survival, the function in granulosa cells is still unclear. An inhibitory pathway indicated by a question mark and leading to decreased cGMP, is inferred from the data shown in Figure 9.

When the activation of targets of LH and EGFR signaling in preovulatory follicles was examined, LH-induced phosphorylation of p38MAPK and MAPK3/1 was found to be partially decreased in *Areg^−/−^ Egfr^wa2/wa2^* and *Egfr^delta/f^ Cyp19-Cre* follicles compared to wild type, suggesting that these pathways are activated both upstream (or in parallel) and downstream of the EGFR (Figure 10). Upstream effects of these pathways on EGF-like growth factor expression and shedding have been reported [35], [36]. Activation of p38MAPK and MAPK3/1 downstream of EGFR is likely to be important for oocyte maturation, as decreased activation of these kinases in the mutant follicles is associated with impaired oocyte maturation. When the impact of impaired EGFR signaling is considered throughout the time course of LH action, our results indicate that LH requires the EGFR for activated MAPK3/1 to be amplified and stabilized in the follicle; recent pharmacological data shows that sustained EGFR activity is required for prolonged MAPK3/1 phosphorylation [37]. Recently, Fan et al. [27] reported the failure of oocytes to mature in mice with disruption of *MAPK3/1* in granulosa cells. Cumulus cells lacking MAPK3/1 failed to respond to AREG in stimulating oocyte GVBD [27]. However, our study and Norris et al [26] show that the MEK inhibitor U0126 is only partially effective in preventing either LH- or AREG-induced oocyte maturation. Although substantially reduced, MAPK3/1 phosphorylation is not completely ablated when the EGF network is disrupted. Also, activation of MAPK3/1 in cumulus cells by recombinant growth and differentiation factor 9 was not sufficient to cause oocyte maturation in cultured COCs [38]. Thus, it is tempting to speculate that oocyte maturation requires EGFR-dependent activation of MAPK3/1. However, activation of MAPK3/1 is necessary but not sufficient for the induction of oocyte meiotic resumption.

Our findings support the novel concept that EGFR transactivation may also be important in terms of spatial propagation of the LH signal in the follicle. A time course of MAPK3/1 phosphorylation in preovulatory follicles clearly shows that phosphorylation is initially confined to the mural granulosa cells lining the antrum, with the exception of the area adjacent to the cumulus cells. Only at later times does phosphorylation occur in the cumulus and adjacent mural granulosa cells. The EGF network plays a crucial role in generating this spatial propagation of signal, because in the *Areg^−/−^ Egfr^wa2/wa2^* follicles, activation of MAPK3/1 in the cumulus is reduced and temporally delayed. Our findings also demonstrate that mural granulosa cells may be heterogeneous in their signaling properties, with a subset of cells retaining a phenotype more similar to cumulus cells than mural granulosa cells. This reinforces the concept that proximity to the oocyte has a profound influence on granulosa cell phenotype, and that oocyte signals can reach beyond the cumulus proper [39] .

Disruption of EGFR signaling in granulosa cells also prevents the LH-induced Cx43 phosphorylation on specific serine residues associated with gap junction closure; decreased Cx43 phosphorylation was observed in mutant follicles as early as 30 min after LH stimulation. Our observations are consistent with recent data showing reduced Cx43 phosphorylation on S262 at 2 h post hCG in whole ovarian lysates from two different mouse models with reduced EGFR activity [31], and inhibition of gap junction closure in response to LH by AG1478 [40]. The phosphorylation and closure of Cx43 gap junctions is dependent on MAPK3/1 [26], but may involve other kinases as well. Here, we show that LH-induced phosphorylation of Cx43 on S262 and S255 is inhibited by the p38MAPK inhibitor, SB202190. Although Cx43 phosphorylation is substantially inhibited in the mutant follicles, p38MAPK and MAPK3/1 phosphorylations are only partially reduced. It is possible that p38MAPK and MAPK3/1 play a synergistic or a hierarchical role.

Data from several studies indicate that pharmacological inhibition of gap junction permeability or mechanical disruption of gap junction connections is sufficient to induce oocyte maturation [23], [24], [25], [26]. However, our study and that of another laboratory [26] have identified conditions where oocyte maturation occurs with gaps still permeable. Norris et al [26] have shown that concentrations of U0126 which completely block Cx43 phosphorylation and preserve dye diffusion between cells do not prevent LH-induced oocyte maturation. In the same vein, we show that AREG can cause oocyte maturation even when Cx43 phosphorylation is prevented. Together, these findings indicate that Cx43 gap closure can be dissociated from oocyte maturation. Thus, Cx43 gap junction closure is sufficient but not indispensable for the induction of oocyte meiotic resumption.

Recent observations have re-proposed a role for cGMP in the control of oocyte meiotic arrest and meiotic maturation [21], [22], [41], [42], [43]. LH causes a profound decrease in cGMP in the follicle and in the oocyte, which in turn should lead to activation of PDE3A. This decrease in cGMP is reproduced by treating follicles with AREG or EREG ([22] and herein). Consistent with the above findings, the LH effect is blocked by the EGFR tyrosine kinase inhibitor AG1478. However, genetic inactivation of *Egfr* does not prevent the LH-dependent decrease in cGMP in the follicle, even though the final concentration in *Egfr^delta/f^ Cyp19-Cre* follicles is twice as high as in the wild type. Interestingly, the effects of AG1478 are absent in the *Egfr^delta/f^ Cyp19-Cre* follicles, a finding that confirms EGFR as the target of AG1478 inhibition. To reconcile the discrepancy between the effect of AG1478 in wild type follicles and the partial cGMP suppression in *Egfr^delta/f^ Cyp19-Cre* follicles, we propose the following hypothesis. In wild type cells, LH suppression of cGMP is mediated predominantly through EGFR activation. However, when EGFR is absent, a secondary pathway independent of EGFR mediates the LH effects and becomes predominant in the *Egfr* null cells. At variance with our results, Norris et al. [40] reported that AG1478 only partially prevents the cGMP decrease. We propose that in these in vitro-matured follicles, the model used by Norris et al [40], the secondary pathway is predominant, whereas in our model using in vivo-matured antral follicles, LH signals predominantly through EGFR. It remains to be determined whether the decrease in cGMP observed in the *Egfr^delta/f^ Cyp19-Cre* follicles is sufficient to promote oocyte maturation. Given the higher cGMP levels, it is possible that in the oocyte, PDE3A is not sufficiently activated to promote maturation. In addition, since Cx43 gap junctions are not phosphorylated and therefore likely open in the *Egfr^delta/f^ Cyp19-Cre* follicles, continuous cGMP diffusion from the somatic cells may contribute to the maintenance of meiotic arrest. Alternatively, the cGMP decrease is per se not sufficient to signal maturation and another positive, EGFR-mediated event is also necessary to induce maturation. Downs et al. [44] have suggested that a positive signal contributes to the induction of meiotic resumption.

The present genetic and pharmacological studies provide insight into the function of EGFR signaling in the somatic compartment of the follicle for the control of the oocyte meiotic cell cycle. A scheme of the multiple pathways involved in LH transactivation of EGFR and downstream signals involved in maturation is reported in Figure 10 and summarizes our findings. The spatial propagation of signals downstream of LH and EGFR within the follicle is likely to be important for the regulation of oocyte maturation and may provide important new information. Disruption of Cx43 phosphorylation in *Egfr^delta/f^ Cyp19-Cre* follicles suggests that these gap junctions are still permeable to positive and/or negative signals such as cGMP. However, cGMP levels still decrease in response to LH in *Egfr^delta/f^ Cyp19-Cre* follicles, suggesting the presence of compensatory regulatory mechanisms. Taken together, our findings indicate that complex, redundant pathways are activated at the time of ovulation and contribute to the control of oocyte reentry into the meiotic cell cycle.
